# *In Vivo* Fluorescence Imaging of Bacteriogenic Cyanide in the Lungs of Live Mice Infected with Cystic Fibrosis Pathogens

**DOI:** 10.1371/journal.pone.0021387

**Published:** 2011-07-07

**Authors:** Seong-Won Nam, Xiaoqiang Chen, Jeesun Lim, So Hyun Kim, Sang-Tae Kim, You-Hee Cho, Juyoung Yoon, Sungsu Park

**Affiliations:** 1 Department of Chemistry and Nano Science, Ewha Womans University, Seoul, Korea; 2 Department of Bioinspired Science, Ewha Womans University, Seoul, Korea; 3 Division of Life and Pharmaceutical Sciences, Ewha Womans University, Seoul, Korea; 4 Department of Pharmacy, CHA University, Gyeonggi, Korea; 5 Mechanobiology Institute (MBI), National University of Singapore (NUS), Singapore, Singapore; Queen Mary University of London, United Kingdom

## Abstract

**Background:**

*Pseudomonas aeruginosa* (PA) and *Burkholderia cepacia* complex (Bcc), commonly found in the lungs of cystic fibrosis (CF) patients, often produce cyanide (CN), which inhibits cellular respiration. CN in sputa is a potential biomarker for lung infection by CF pathogens. However, its actual concentration in the infected lungs is unknown.

**Methods and Findings:**

This work reports observation of CN in the lungs of mice infected with cyanogenic PA or Bcc strains using a CN fluorescent chemosensor (4′,5′-fluorescein dicarboxaldehyde) with a whole animal imaging system. When the CN chemosensor was injected into the lungs of mice intratracheally infected with either PA or *B. cepacia* strains embedded in agar beads, CN was detected in the millimolar range (1.8 to 4 mM) in the infected lungs. CN concentration in PA-infected lungs rapidly increased within 24 hours but gradually decreased over the following days, while CN concentration in *B. cepacia*-infected lungs slowly increased, reaching a maximum at 5 days. CN concentrations correlated with the bacterial loads in the lungs. *In vivo* efficacy of antimicrobial treatments was tested in live mice by monitoring bacteriogenic CN in the lungs.

**Conclusions:**

The *in vivo* imaging method was also found suitable for minimally invasive testing the efficacy of antibiotic compounds as well as for aiding the understanding of bacterial cyanogenesis in CF lungs.

## Introduction

Cystic fibrosis (CF) is the most common genetic disease in Caucasians [1]. Its main clinical features are excessive mucus production, airway obstruction, chronic airway infection and chronic inflammation, leading to severe bronchiectasis, irreversible lung damage and respiratory failure. *Pseudomonas aeruginosa* (PA) is the most common pathogen and is detected in 60–80% of patients' cultures according to age [2]. PA releases virulence factors that induce pulmonary deterioration in the lungs of CF patients [3], including exotoxin A, elastase, LasA protease and pyocyanin [4]. *Burkholderia cepacia* complex (Bcc), a group of at least nine species including *B. cepacia*, is more dangerous to CF patients than PA [5]. Bcc infection leads to the rapid deterioration of a patient's condition, termed ‘cepacia syndrome’, and approximately 20% of these patients die from high fevers, bacteremia and severe necrotizing pneumonia [6]. Bcc's innate multi-drug resistance to antibiotics is often associated with the high mortality rates [3]. Bcc produces a variety of virulence factors, such as proteases, lipases, hemolysins and exopolysacchride (EPS) [3].

Most PA and Bcc strains are capable of producing cyanide (CN) [7], [8], which binds ferric iron and inhibits the function of cytochrome c oxidase in mitochondria. The lungs of CF patients are typically in a microaerobic condition, where cyanogenic pathogens survive through anaerobic respiration that can increase CN production [7], [9]. Although studies have not yet determined whether CN alone aggravates the conditions of CF patients, growing evidence suggests that it may be a predictor of PA infection in the CF lung [10], [11]. CN has been detected up to 130 µM in the sputa of CF and non-CF bronchiectasis patients infected with PA [10] and has not been detected in the sputa of patients without PA infection [10], [11]. However, monitoring the amount of CN produced by microorganisms in the lung is not easy because sputum samples are not easily obtained from either pediatric patients or small animals, such as mice.

While conventional imaging methods, such as X-ray and MRI (magnetic resonance imaging) show the pathological state of the infected organs, *in vivo* molecular imaging [12] offers noninvasive insight into living organisms and provides spatial and temporal information of disease-related changes in the body. Optical molecular imaging [13], [14] can detect light emitted from chemiluminescent or fluorescent probes and is relatively safe, cheap and easy to handle compared with MRI or nuclear imaging. Fluorescent chemosensors selectively bind to ions [15] or metals [16], changing their emission or excitation/emission profiles. This property has been exploited to study cell physiologies, such as heavy metal transitions [16]. CN specific chemosensors were developed [17]–[20], but their application was limited to detecting CN in environmental samples, such as water. Recently, we have successfully visualized CN in the nematode *Caenorhabditis elegans* using chemosensors [21], [22].

In this study, CN production was monitored and quantified in the lungs of mice infected with PA or *B. cepacia* strains. For chronic infections, mice were intratracheally infected with these strains embedded in agar beads [23], [24]. An “off-on” type CN chemosensor was directly injected into the lungs before the whole animal imaging. The effects of antimicrobial compounds on CN production were visualized as well.

## Materials and Methods

### Synthesis and fluorescent assays of the CN sensor

The sensor was synthesized by a previously reported method [25]. Fluorescein (4 g, 12 mmol), 10 mL CHCl_3_, 6 mL CH_3_OH and 0.06 g 15-crown-5 were placed in a 100 mL flask to which 20 g 50% NaOH solution was carefully added at 55°C. The mixture was stirred for 5 h. After cooling, it was acidified with 10 M H_2_SO_4_. The precipitates were collected and dried in vacuum. Finally, the white solid product (142 mg, yield 3.0%) was obtained by chromatography on a silica gel column using CH_3_COOCH_2_CH_3_/CH_2_Cl_2_ (5∶95, v/v) as eluent. Fluorescent assays used test solutions of the sensor (3 µM) and a various concentration of the sodium salts of different anions (CN^−^, H_2_PO_4_^−^, HSO_4_^−^, NO_3_^−^, CH_3_CO_2_^−^, F^−^, Cl^−^, Br^−^ and I^−^) in HEPES buffer (0.01 M, pH 7.4, 33% CH_3_CN). Fluorescence emission spectra were obtained using a RF-5301/PC spectrofluorophotometer (Shimadzu Co.).

### Bacterial strains and CN determination in the bacterial culture

All PA strains were grown overnight in LB medium (BD) at 37°C and 220 rpm. The mutant required gentamicin (Sigma Chemical Co.). Supernatants were harvested at the optical density, OD_600_ = 0.9 and CN was assayed using Spectroquant® CN detection kits (Merck).

*B. cepacia* (KCTC 2966) was obtained from the Biological Resource Center (Daejeon, Korea). It was grown in 470 bacteria culture medium (Sigma-Aldrich) at 30°C and 220 rpm. The supernatant was harvested at OD_600_ = 2.6 and CN was assayed with the kit. To determine the amount of CN produced from *B. cepacia* biofilm, 12 mL of a 1∶10 dilution of the culture (OD_600_ = 0.5) was inoculated in a Petri dish containing 48 g of glass beads and CN was trapped in a separate dish containing 4 M NaOH as described previously [8]. CN trapped in NaOH was assayed using the CN detection kits (Merck).

### Introduction of NaCN to the lungs

6-week-old female BALB/c nude mice (17 to 19 g) were purchased from Orientbio Inc. (Seongnam, Korea). They were treated in accordance with the university's guidelines. The mice were anesthetized by intraperitoneal (i.p.) injection with 10 µL (each 0.25 mg tiletamine, zolazepam) zoletil®50 (Virbac, Carros, France) using an insulin syringe (Shinchang medical co., Gumi, Korea). Then, 40 µL of various concentrations of NaCN (0.1 mM–1 M) were injected into their lungs using a syringe.

### Infection with PA or *B. cepacia*

50 µL of bacterial suspension in agar beads containing approximately 4.5×10^6^ CFU was inoculated into the anesthetized mice via intratracheal instillation [23], [24] using a 24-gauge catheter (BD).

### *In vivo* imaging

A multimodal-imaging system IS4000MM (Kodak) was used at 465 nm and an emission at 535 nm. After 40 µL of the CN sensor (1 mM) was injected into the lungs of the anesthetized mice, the animals were exposed for 10 sec. Two rectangular regions of interest (ROI) with dimensions of 280 pixels (width)×222 pixels (height) were manually drawn on each animal image with a dimension of 800 pixels (width)×1,400 pixels (height) using the program Image J (NIH, USA). One of the regions was the infectious or affected area, covering the lobes, for the signal intensity; the other ROI, outside the infectious area, was for background intensity. The value of the background intensity was subtracted from the signal intensity in order to obtain the normalized signal intensity from each image.

### *Ex vivo* imaging of CN and biofilm in the lungs

20 µm thick lung samples on glass slides [26] were incubated with the sensor (2 µM) and TRITC (tetramethyl-rhodamine-isothiocyanate)-labeled concanavalin A (50 µg/mL) (Sigma-Aldrich) for 5 min. The images were obtained using a confocal laser scanning microscope (CLSM) (LSM510, Carl Zeiss) with 488 nm excitation and 505–530 nm emission filters for the sensor and 543 nm excitation and 560–615 nm emission filters for concanavalin A-TRITC at 400×magnification.

### *In vivo* efficacy testing of antimicrobial compounds and CN antidote

Ceftazidime, ciprofloxacin and patulin were purchased from Sigma-Aldrich. PA14-infected mice were treated with either ceftazidime [26] (200 mg/kg), through tail vein (i.v.) injection, or ciprofloxacin [26] (30 mg/kg), through oral administration, at 18 h after infection. Patulin [27] (50 µg) was injected into the mice intraperitoneally (i.p.) once a day for 3 days. CN in the lung was imaged 6 h. after the treatments. The mice infected with *B. cepacia* were treated with either ceftazidime (200 mg/kg, i.v.) or patulin (50 µg, i.p.) 5 days after infection. Hydroxocobalamin (B12a) [28] was purchased from Sigma-Aldrich. B12a (70 mg/kg) was injected directly into the mouse lungs that had been either injected with 0.1 M NaCN or infected with PA14.

### Bacterial load determinations

The lungs that were obtained from the sacrificed mice were homogenized in 1 ml of sterile PBS, plated in duplicates on BBL™ eosin methylene blue agar (BD) and incubated at 37°C for 18 h prior to CFU determinations.

### Cell death in the infected lung tissue

20 µm thick fresh frozen sections from the mice that were previously either injected NaCN or infected with PA14 were incubated on a 45°C hot plate for 2 h and then washed sequentially with 100%, 95%, 70% ethanol and PBS. Samples were fixed with 3.7% buffered formaldehyde for 10 min. and washed with PBS. A TACS® 2 TdT-Fluor *in situ* apoptosis detection kit (Trevigen, Gaithersburg, MD, USA) was used to examine whether bacteriogenic CN induced apoptosis in the infected lungs. As a positive control, TACS-nuclease was added to tissue sections, which induced apoptosis. To examine necrotic cells, tissue sections were counterstained with 1.5 µM propidium iodide (Invitrogen).

### Statistics

The data are reported as dot blots including every data points of n independent mice and independent experiments. Significant difference was calculated with one-way analysis of variance (ANOVA) was with Bonferroni post-tests and defined as *p*<0.05 using an unpaired two-tailed *t* test [29] (Graphpad, La Jolla, CA, USA).

## Results

### Sensitivity and selectivity of CN sensor in solution

**Figure 1:**
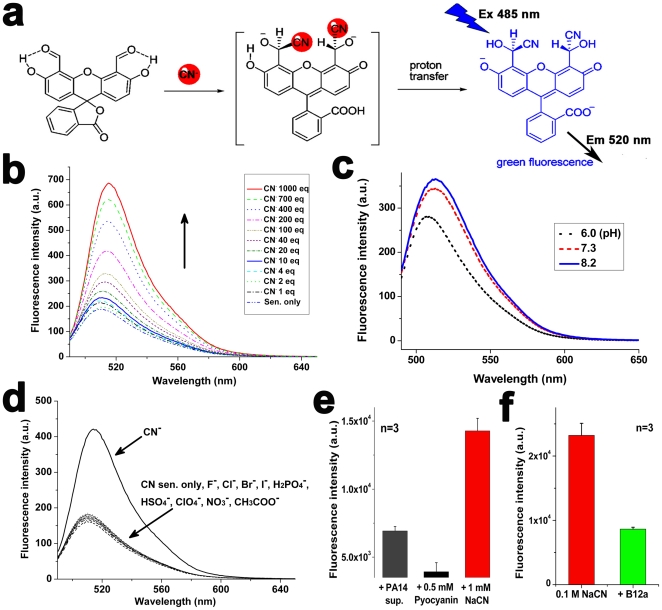
Proposed mechanism of CN sensing with synthesized fluorescein dialdehyde as the CN sensor and the characterization of the sensor in aqueous solution. (a) CN ions selectively attack carbonyl groups of fluorescein dialdehyde and the phenol protons can be transferred to the developing alkoxide anion, resulting in the enhancement of fluorescence. (b) Fluorescence spectra of the sensor (3 µM) with different concentrations of NaCN (1–1,000 eq.) in CH_3_CN/HEPES at room temperature. (c) Effect of pH on fluorescence spectra of the sensor (3 µM) in the absence of CN at room temperature in 100% water. (d) Fluorescence spectra of the sensor (3 µM) with various anions (200 eq.) in CH_3_CN/HEPES at room temperature. (e) Fluorescence intensity of the CN sensor (1 mM) with either supernatant obtained from PA14 culture in LB (O.D. = 3.3, pH 7.4), PBS (pH 7.4) containing pyocyanin (0.5 mM) or NaCN (1 mM). The graph represents the mean ± standard deviation of three independent experiments. (f) Fluorescence intensity of the CN sensor (1 mM) with NaCN (0.1 M) in PBS (pH 7.4) in the absence or presence of B12a (2.5 mM). The graph represents the mean ± standard deviation of three independent experiments.

The title sensor 4′,5′-fluorescein dicarboxaldehyde (**Fig. 1a**), reported as an intermediate in previous work [30], was synthesized and used as a CN sensor for the first time in this study to fluorescently observe CN in lungs. The sensing mechanism involved CN acting as a selective nucleophilic agent for *o*-hydroxy-substituted aromatic aldehyde [31] and could thus attack the carbonyl group, which was activated by phenol protons (**Fig. 1a**). Protons were then transferred from phenol hydrogen to the developing alkoxide anion, resulting in the fluorescence enhancement (**Fig. 1a**). In HEPES buffer (0.01 M, pH 7.4, 33% CH_3_CN), the enhancement of fluorescence depended on CN concentration (**Fig. 1b**). In 100% water, there was no significant change in fluorescence intensities when pH was adjusted from 6.0 to 8.2 (**Fig. 1c**). The CN sensor selectively reacted with CN^−^ ions among the various other anions in HEPES buffer, such as F^−^, Cl^−^, Br^−^, I^−^, H_2_PO_4_^−^, HSO_4_^−^, ClO_4_^−^, NO_3_^−^ and CH_3_COO^−^ (**Fig. 1d**). The sensor did not react with PA14 supernatant or pyocyanin, a toxin produced by PA (**Fig. 1e**). Its reactivity with CN interfered with B12a [28], known to bind strongly with CN (**Fig. 1f**). Taken together, these results indicate that the sensor specifically binds to CN, enhancing its fluorescence in aqueous solution.

### *In vivo* fluorescence imaging of exogenous CN in mice

The sensor was prepared in dimethyl sulfoxide (DMSO), known to have low toxicity [32]. Before applying the CN sensor into mouse, its toxicity was tested by daily injecting 40 µL of the sensor (1 mM) into the lungs of three mice for 10 days. The direct injection of the sensor into the lobes did not cause vomiting or death. During the period, the injected mice did not show any differences in behavior relative to controls that had not been injected with the sensor (data not shown).

**Figure 2:**
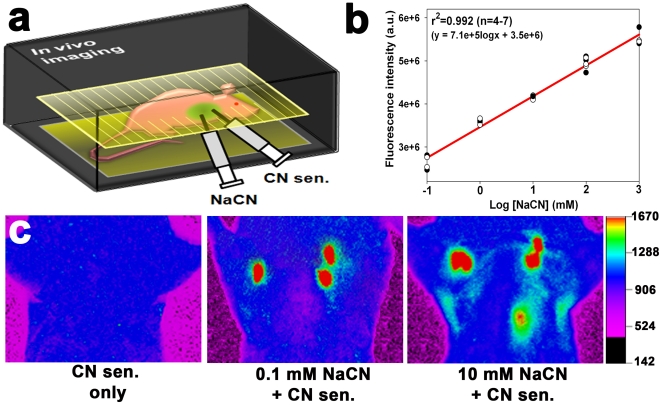
*In vivo* imaging of CN in the lungs of live mice using the CN sensor. (a) Experimental scheme for the *in vivo* imaging in the mouse lungs injected with NaCN. (b) Changes in fluorescence intensity as a function of NaCN concentration in the murine lungs. Four to seven mice were used per treatment, and each dot in the graph represents a background-subtracted fluorescence intensity obtained from a mouse in each treatment group after subtracting background fluorescence intensity. Total twenty seven mice were used. (c) *In vivo* images of CN in the mouse lungs after injection with NaCN. The fluorescence intensity is shown in arbitrary units.

In the following experiments, 40 µL of various concentrations of NaCN (0.1 mM–1 M) and 40 µL of the CN sensor (1 mM) were injected into anesthetized mice (**Fig. 2a**). Whole animal imaging system was used to determine whether the sensor was capable of visualizing the exogenous CN in the lungs. Strong fluorescence was observed in lungs injected with NaCN (0.1 mM or higher), whereas the fluorescence was not observed in the lungs without NaCN (**Fig. 2c** and **Figure S1**), indicating that the sensor selectively reacted with CN in the lungs. Region of interest (ROI) analyses showed that the sensor's fluorescence intensity the lungs exhibited a linear response with respect to the concentration of injected NaCN (**Fig. 2b**). The correlation coefficient of the linear regression was 0.992 for 4 to 7 independent measurements, and the detection limit was 0.1 mM NaCN (*p* = 0.0026). The reaction of the sensor with CN in mice was inhibited by B12a (**Figure S2**).

### Short-term *in vivo* imaging of biogenic CN in the lungs of mice infected with PA or *B. cepacia*

**Figure 3:**
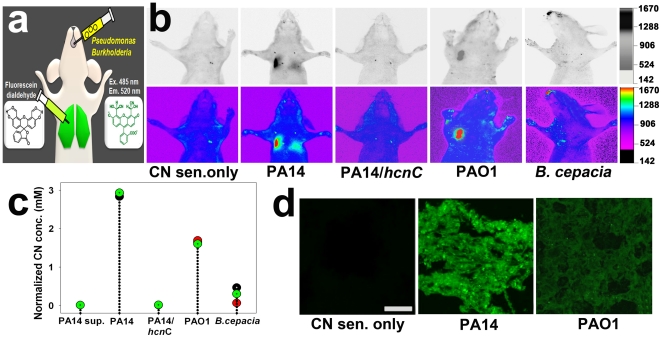
*In vivo* and *ex vivo* images of bacteriogenic CN in the lungs of live mice infected with PA or *B. cepacia* strains. (a) Experimental scheme for the *in vivo* imaging of bacteriogenic CN in the murine lungs. (b) *In vivo* images of CN in the lungs at 18 h. after infection. The images in the upper and lower panels are inverted fluorescence images and their corresponding reconstructed color images, respectively. (c) Quantification of the fluorescence intensity in the lungs of the infected mice. Every three mice were used per treatment and each dot in the graph represents a background-subtracted fluorescence intensity from a mouse in each treatment group. ANOVA with Bonferroni post-tests (*p*<0.0001, n = 15). (d) CLSM images of the cryo-sectioned lung tissue. Before imaging, 20 µm thick lung tissue samples were incubated with the sensor (2 µM) for 5 min. Scale bar = 50 µm.

To chronically infect the mouse lungs, mice were intratracheally infected with PA or *B. cepacia* strains embedded in agar beads [23], [24] (**Fig. 3a**). As shown in **Fig. 3b**, stronger fluorescence was observed in PA14 [33] - or PAO1 [34] -infected lungs, compared with either PA14 *hcnC* mutant [35] -, *B. cepacia*-infected or uninfected lungs (CN sensor only or PA14 supernatant), when 40 µL of the CN sensor (1 mM) was directly injected into the lungs at 18 h. after the infection. The dose-response curve (**Fig. 2b**) of the fluorescence signals in the lungs was used to estimate CN concentration in the lungs. Surprisingly, both PA strains produced millimolar concentrations (1.8 to 2.9 mM) of CN in the lung (**Fig. 3c**). Interestingly, the wild type PA14 produced stronger signals than wild type PAO1. These *in vivo* data were verified by *ex vivo* imaging results (**Fig. 3d**), which exhibited a similar tendency in fluorescence intensity. The reaction of the sensor with CN in PA14-infected lungs was inhibited by B12a (**Figure S2**). Although intranasal application of the sensor appeared to be least invasive, it was not used in this study as it caused vomiting, and thus required a high volume (*ca.* 200 µL) of the sensor in the infected mice (**Figure S3**). In contrast, direct injection of the sensor in the lung did not cause vomiting and 40 µL of the sensor (1 mM) generated the highest signal to noise ratio to bacteriogenic CN (**Figure S4**).

### Long-term *in vivo* imaging of biogenic CN in lungs of mice infected with PA or *B. cepacia*

**Figure 4:**
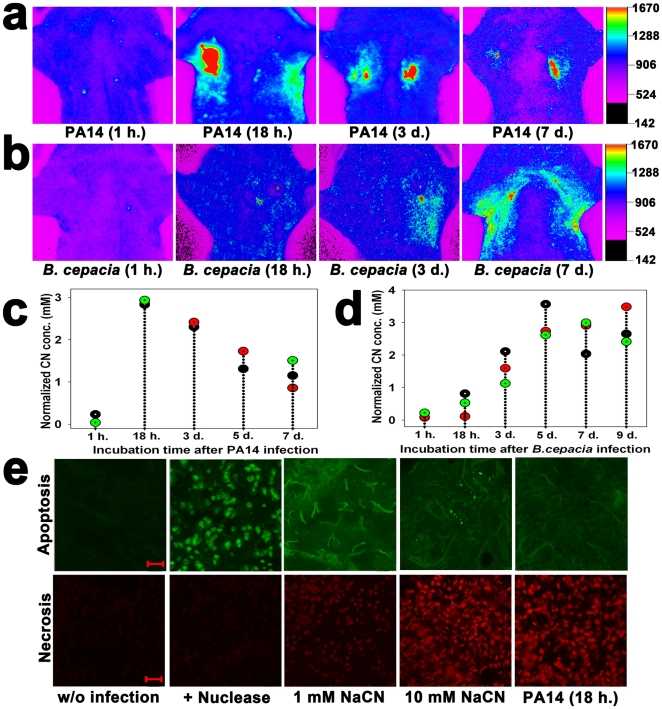
Long-term monitoring of CN production in the lungs of mice infected with PA14 or *B. cepacia* and tunnel assays of the lung sections. (a) *In vivo* images of CN at various incubation times (1 h.–7 days) after PA14 infection. (b) *In vivo* images of CN at various incubation times (1 h.–7 days.) after *B. cepacia* infection. (c) Quantification of the fluorescence intensity in the PA14-infected lungs. Each dot represents a background-subtracted fluorescence intensity from a mouse in each treatment group, and every three mice were given each treatment (total n = 15). (d) Quantification of the fluorescence intensity in the *B. cepacia*-infected lungs. Each dot represents a background-subtracted fluorescence intensity from a mouse in each treatment group, and every three mice were given each treatment (total n = 18). (e) CLSM images of the 20 µm thick cryo-sectioned lung tissue samples after tunnel assay using a TACS® 2 TdT-Fluor *in situ* apoptosis detection kit and incubation with 1.5 µM propidium iodide. TACS-nuclease treatment before tunnel assay was used as a positive control. The sections from the murine lungs injected with 1, 10 mM NaCN or infected with PA14 for 18 h. Scale bar = 20 µm.

*In vivo* imaging was used to monitor CN production in the lungs of live mice infected with either wild type PA14 or *B. cepacia* strains for up to 9 days. In PA14-infected lungs, CN concentration rapidly increased within 24 hours but gradually decreased over the following days (**Fig. 4a,c**). In contrast, in *B. cepacia*-infected lungs, CN concentration slowly increased, reaching a maximum at 5 days, after which it remained constant (**Fig. 4b,d**). Irrespective of this difference in CN production pattern between PA14 and *B. cepacia* strains, both strains were able to continuously produce millimolar concentrations of CN in the lung even 7 days after infection (**Fig. 4c,d**), indicating that the lungs were chronically infected with the strains.

Tunnel assay showed that the apoptotic signal was stronger than the necrotic signal when lungs were injected with 1 mM NaCN, while the reverse occurred with injection of 10 mM NaCN (**Fig. 4e**), indicating that the mode of cell death depended on CN concentration. Lung sections of mice infected with PA14 for 18 h. also revealed stronger necrotic signals (**Fig. 4e**), implying that the CN concentration in the PA14-infected lungs was higher than 1 mM. CN concentration-dependent death mode has been reported in cultured primary rat cortical cells [36]. The *ex vivo* imaging results show that the bacteria in the lungs produced a large amount of CN as well as EPS (**Figure S5**), a major constituent of bacterial biofilm.

### *In vivo* efficacy of antimicrobial compounds against PA and *B. cepacia* infections

**Figure 5:**
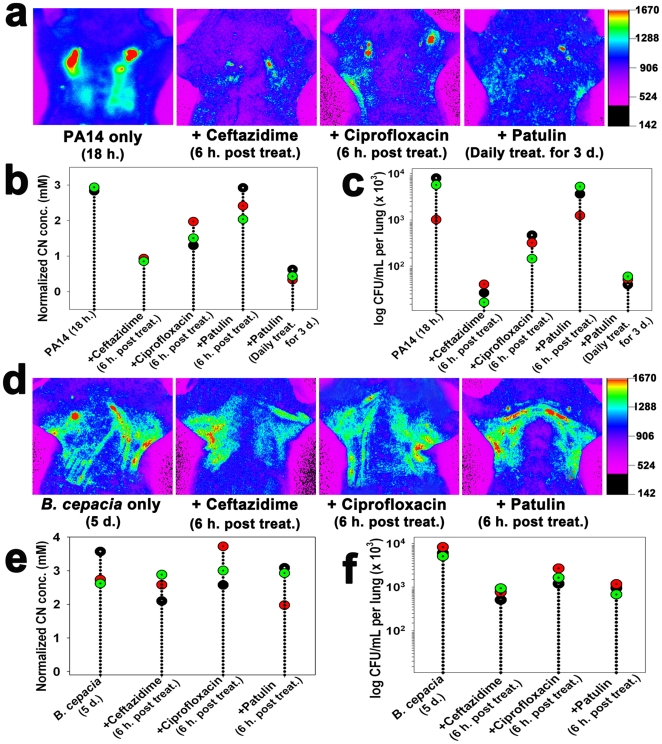
The effects of antimicrobial treatments on CN production in infected lungs. (a) *In vivo* images of CN in the lungs of PA14-infected mice treated with antibiotics. 18 h. post infection either 200 mg/kg ceftazidime was injected in the tail vein (i.v.) or 30 mg/kg ciprofloxacin was orally administered using a 24-gauge catheter (p.o.). 50 µg patulin was treated intraperitoneally (i.p.) daily for 3 days. The sensor was then directly injected into the lungs at 6 h. after the antibiotic treatment. (b) Quantification of the fluorescence intensity in the PA14-infected lungs treated with antibiotics. Each dot represents a CN concentration from a mouse in each treatment group, and every three mice were given each treatment (total n = 15). ANOVA with Bonferroni post-tests (*p*<0.0001). The efficacy of each antibiotics against PA14 infection was determined using Student's unpaired t-test: ceftazidime, *p*<0.0001: ciprofloxacin, *p* = 0.0029: patulin for 3 days, *p*<0.0001. (c) Reduced bacterial loads in the PA14-infected lungs after each antimicrobial treatment. The PA14-infected lungs were excised from the mice at 6 h. after the antibiotic treatment and CFUs in the excised lungs were determined. Each dot represents the mean CFU of four different lung samples from independent mice and three independent experiments were performed per treatment. ANOVA with Bonferroni post-tests (*p*<0.0001). (d) *In vivo* images of CN in *B. cepacia*-infected lungs treated with antibiotics. 200 mg/kg ceftazidime was injected into the tail vein, 30 mg/kg ciprofloxacin was orally administered, and 50 µg patulin was intraperitoneally administered (i.p.) daily for 5 days after infection. (e) Quantification of the fluorescence intensity in the lungs of the *B. cepacia*-infected mice treated with antibiotics. Each dot represent a CN concentration from a mouse in each treatment group and three mice were used per each treatment (total n = 12). ANOVA with Bonferroni post-tests (*p* = 0.8760). (f) *B. cepacia* loads in the lungs treated with the antibiotics. Each data point represents the mean CFU of four different lung samples from independent mice and three independent experiments were performed per treatment. ANOVA with Bonferroni post-tests (*p* = 0.1040).

When mice infected with wild type PA14 were treated with either ceftazidime (200 mg/kg) or ciprofloxacin (30 mg/kg) [26] at 18 h. after infection, both CN production (**Fig. 5a,b**) and bacterial load (**Fig. 5c**) in the lungs significantly decreased (*p*<0.0001), indicating that the antibiotic treatments were effective against the lung infection. Both CN production and bacterial loads significantly decreased (*p*<0.0001) when mice infected with PA14 for 18 h were treated with patulin (50 µg) [27], a fungal toxin that inhibits bacterial communication, for 3 days intraperitoneally, indicating that the toxin was capable of inhibiting the infection *in vivo*.

In contrast, the individual antibiotic treatments were not effective against *B. cepacia* infection. Neither CN production nor bacterial loads significantly decreased in the infected lungs (**Fig. 5d–f**), indicating that the *B. cepacia* strain was resistant to all the antibiotic treatments.

## Discussion

The CN sensor was highly specific for CN both *in vitro* and *in vivo*. Only CN induced fluorescence enhancement of fluorescein dicarboxyaldehyde among various anions in aqueous solution (**Fig. 1d**). This selectivity was attributable to the specific reaction between the aldehyde group of the sensor and CN (**Fig. 1a**). The aldehyde group allows intramolecular hydrogen bonding, thereby facilitating nucleophilic attack from CN. The specificity of the sensor to CN in aqueous solution was further verified by results showing that its fluorescence response to CN was not affected by either PA14 supernatant or pyocyanin (**Fig. 1e**), while it was reduced by a CN antidote, B12a [28] (**Fig. 1f**). Similarly, *hcnC* mutant strain did not induce any fluorescent signal in the infected lung (**Fig. 3b,c**) and B12a was able to interfere with the reaction of the sensor with CN in the lung (**Figure S3**), demonstrating the *in vivo* specificity of the sensor to CN. It is expected that physiological pH (6.9) in the lung [37] would not affect the fluorescence response of the sensor, as there was no significant difference in the fluorescence intensities of the sensor over the pH range 6.0–8.2 (**Fig. 1c**). Using the sensor and whole animal imaging, concentrations as low as 0.1 mM NaCN were observed in the lung (**Fig. 2**).

The presence of CN at high concentrations (1.8 to 4 mM) allowed visualization of bacteriogenic CN in the lungs of live mice infected with PA or *B. cepacia* strains. These strains in the lung produced more CN than they did in the planktonic culture (**Table S1**). CN production by PA or *B. cepacia* strains might be enhanced by a microaerophilic condition in the infected lungs, where HCN synthases are highly activated [7], [9]. The microaerophilic condition in the lung might have been contributed by biofilm formation (**Figure S5**). Especially, *B. cepacia* is known to produce CN only when it establishes mature biofilm [8]. This may explain why it did not produce detectable amounts of CN in the lung until 2–3 days after infection (**Fig. 4b**). Once it started to produce CN, its CN production was continued even at 9 day after infection (**Fig. 4b,d**). These results suggest that CF lung, which is microaerophilic, is suited for the cyanogenic bacteria to produce CN.

Cell death by necrosis (**Fig. 4e**) indicates the presence of millimolar CN in the infected lungs. It was shown that CN at 1 mM or higher concentrations caused necrosis in epithelial lung cells [38]. Other major virulence factors including pyocyanin in PA are known to apoptosis in the lung [39], [40]. The CN concentration (2.9 mM) in the murine lungs infected with PA14 can be converted to be 1.7 mg/kg, a fifth of the lethal dose (8.4 mg/kg) in mice [41]. At this concentration, CN was capable of inhibiting cytochrome oxidase activity by up to 15%, causing unconsciousness in the mice for 10 to 60 min [42]. In fact, many of the infected mice vomited and did not eat for two days, some died (data not shown). Necrosis in the lung has been reported to enhance inflammation at the site of infection [43]. These data suggest that, along with other virulence factors, biogenic CN in the lungs may have detrimental effects in CF patients.

CN concentration in the lungs was clearly related to the respective bacterial loads (**Fig. 5**), suggesting that bacteriogenic CN acted as a biomarker of the cyanogenic bacteria in the lungs. Information about the effective route of antibiotic administration, as well as the *in vivo* efficacy of the antibiotics, was obtained from this correlation (**Fig. 5**). PA14 was susceptible to β-lactam (e.g., ceftazidime) or fluoroquinolone (e.g., ciprofloxacin) antibiotics as well as patulin (**Fig. 5a–c**). Patulin inhibits quorum sensing [44], which allows bacteria to produce responses such as virulence factors or biofilm at a critical cell density. PA has been reported to be effectively cleared from mice by treatment with patulin [27]. In contrast, *B. cepacia* was resistant to all the antimicrobial treatments (**Fig. 5d–f**). This is possible because it has β-lactamase as well as antibiotic efflux system [45]. These results suggest that the current CN imaging method is suitable for testing the *in vivo* efficacy of antibacterial compounds and CN inhibitors. The method has several advantages in testing antibiotic susceptibility of PA in vivo over the *in vivo* imaging method using luciferase-tagged isolates [46], [47]. Unlike the latter, the former does not require a construction of a strain carrying the luciferase gene and its fluorescence signal is not affected by exhaustion of flavin mononucleotide, which is a substrate of luciferase [46], [47].

Collectively, these results suggest that the chemosensor-based *in vivo* imaging method is useful for fast and convenient animal testing of drug efficacy against the PA and *B. cepacia* infections as well as understanding cyanogenesis in CF lungs. In future, a CFTR-deficient murine model [48] could be used to elucidate the pathological role of biogenic CN in CF patients.

## Supporting Information

Figure S1 Representative *in vivo* inverted fluorescence images and their corresponding reconstructed color images (top and bottom respectively) of the mice that were injected with various concentrations of NaCN (0.1 mM–1 M). Then, 40 uL of the CN sensor (1 mM) were injected into the lung of the mice for *in vivo* imaging.
(DOCX)

Figure S2 Inhibition of exogenous and bacteriogenic CN by a CN antidote, hydroxocobalamin (B12a). The mice were either injected with 40 µL of NaCN (0.1 M) or infected with PA14 for 18 h. Then, 40 µL of B12a (70 mg/kg, 24.9 mM) were additionally injected into the mice before the imaging.
(DOCX)

Figure S3 *In vivo* imaging of CN in the lung of PA14-infected mice using the cyanide sensor with the intranasal application. (a) A 24-gauge catheter containing 50 µL of the cyanide sensor (1 mM) was introduced into the nostril of the mouse that was not infected with PA14 (control). 50–200 µL of the CN sensor (1 mM) was injected into the lung of PA14-infected mice through the intranasal application at 18 h. post infection. (b) Changes in the fluorescence intensity as a function of the volume (50 to 200 µL) of the CN sensor that was introduced through the intranasal application into the lungs of the mice infected with PA14. All of the data are given as the mean ± s.d. of n independent measurements.
(DOCX)

Figure S4 Effect of the volume of the CN sensor on i*n vivo* cyanide imaging. (a) Various volumes (0–100 µL) of the CN sensor (1 mM) were injected into the lung of mice infected with PA14. The images in the upper and lower panels are the inverted fluorescence images and their corresponding reconstructed color images, respectively. (b) Changes in the fluorescence intensity as a function of the injected volume of the cyanide sensor in the lung of mice infected with PA14. (c) Signal to noise (S/N) ratio of the fluorescence signal in the mouse lungs with respect to the injected volume of the cyanide sensor. The S/N ratio was defined as the ratio of the fluorescent signal value in the mouse lung infected with PA14 to the signal value without the PA14 infection. All of the data are given as the mean ± standard deviations (s.d.) of n independent measurements.
(DOCX)

Figure S5 *Ex vivo* imaging of CN and biofilm in the lungs of the mice infected with PA14 or *B. cepacia* strains for various times. CLSM images of the 20 µm thick cryo-sections incubated with 2 µM CN sensor for 5 min and 50 µg/mL concanavalin A-TRITC for 5 min. The surface plot image of merged images was obtained using Imaris™ program. Scale bar −20 µm.
(DOCX)

Table S1 CN production in liquid and solid cultures and murine lungs by PA and *B. cepacia* strains. CN in the liquid cultures was determined using the Spectroquant® method* in triplicate (at OD_600_ = 0.9 for PA, 2.6 for *B. cepacia*). CN in the solid cultures containing glass beads were trapped in 4 M NaOH and measured using the method described above. Means ± standard deviations of three independent experiments are shown (n. d. = not detected). Biogenic CN in the lungs previously infected with PA and *B. cepacia* was determined from the images using a multimodal-imaging system (IS4000MM, Kodak). The standard curve (**Fig. 2b**) was used to determine the concentration of biogenic CN in the lungs infected with either PA or *B. cepacia*. *Detailed description in the methods.
(DOCX)
