# Supplementary material for: In Vivo Fluorescence Imaging of Bacteriogenic Cyanide in the Lungs of Live Mice Infected with Cystic Fibrosis Pathogens
Source: PLoS One. 2011 Jul 7;6(7):e21387. doi: 10.1371/journal.pone.0021387 (PMC3131278; doi:10.1371/journal.pone.0021387)
Supplement: Figure S1 — Representative in vivo inverted fluorescence images and their corresponding reconstructed color images (top and bottom respectively) of the mice that were injected with various concentrations of NaCN (0.1 mM–1 M). Then, 40 uL of the CN sensor (1 mM) were injected into the lung of the mice for in vivo imaging. (DOCX) [file pone.0021387.s001.docx]

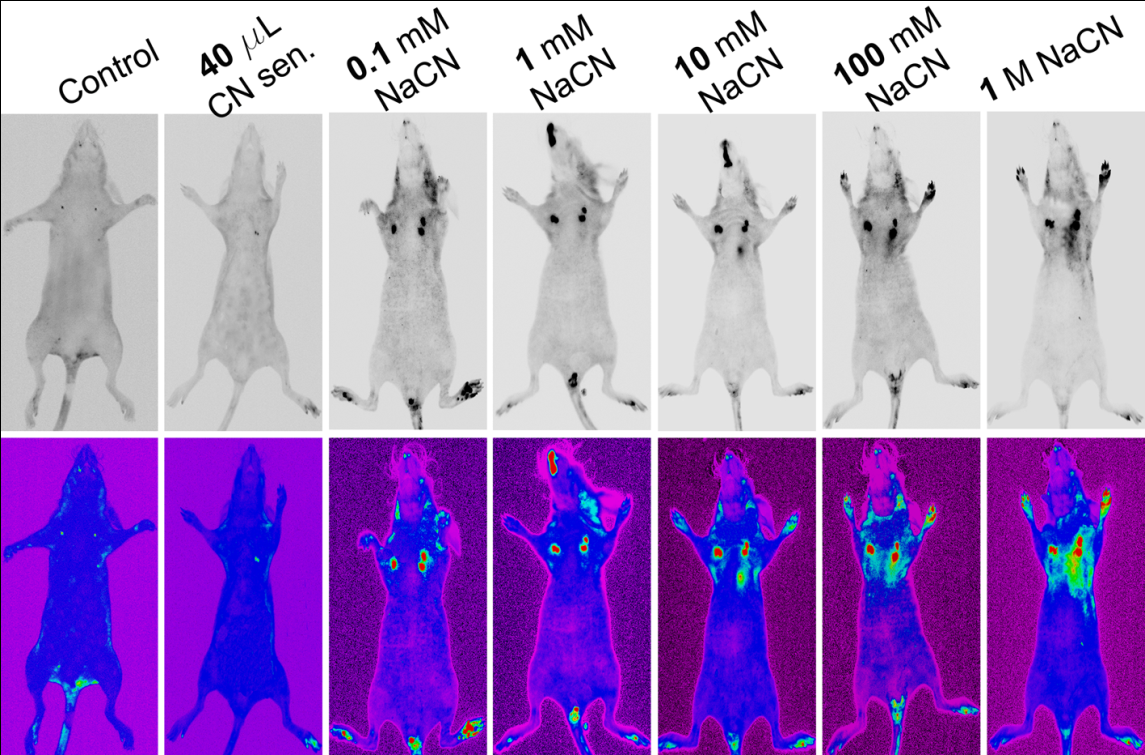


**Figure S1** Representative *in vivo* inverted fluorescence images and their corresponding reconstructed color images (top and bottom respectively) of the mice that were injected with various concentrations of NaCN (0.1 mM – 1 M). Then, 40 uL of the CN sensor (1 mM) were injected into the lung of the mice for *in vivo* imaging.
