# Supplementary material for: In Vivo Fluorescence Imaging of Bacteriogenic Cyanide in the Lungs of Live Mice Infected with Cystic Fibrosis Pathogens
Source: PLoS One. 2011 Jul 7;6(7):e21387. doi: 10.1371/journal.pone.0021387 (PMC3131278; doi:10.1371/journal.pone.0021387)
Supplement: Figure S2 — Inhibition of exogenous and bacteriogenic CN by a CN antidote, hydroxocobalamin (B12a). The mice were either injected with 40 µL of NaCN (0.1 M) or infected with PA14 for 18 h. Then, 40 µL of B12a (70 mg/kg, 24.9 mM) were additionally injected into the mice before the imaging. (DOCX) [file pone.0021387.s002.docx]

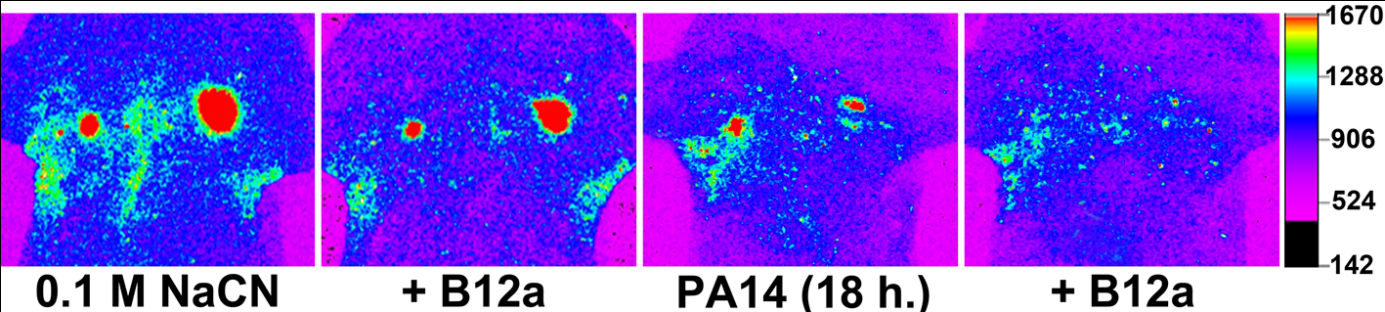


**Figure S2** Inhibition of exogenous and bacteriogenic CN by a CN antidote, hydroxocobalamin (B12a). The mice were either injected with 40 μL of NaCN (0.1 M) or infected with PA14 for 18 h. Then, 40 μL of B12a (70 mg/kg, 24.9 mM) were additionally injected into the mice before the imaging.
