# Supplementary material for: In Vivo Fluorescence Imaging of Bacteriogenic Cyanide in the Lungs of Live Mice Infected with Cystic Fibrosis Pathogens
Source: PLoS One. 2011 Jul 7;6(7):e21387. doi: 10.1371/journal.pone.0021387 (PMC3131278; doi:10.1371/journal.pone.0021387)
Supplement: Figure S3 — In vivo imaging of CN in the lung of PA14-infected mice using the cyanide sensor with the intranasal application. (a) A 24-gauge catheter containing 50 µL of the cyanide sensor (1 mM) was introduced into the nostril of the mouse that was not infected with PA14 (control). 50–200 µL of the CN sensor (1 mM) was injected into the lung of PA14-infected mice through the intranasal application at 18 h. post infection. (b) Changes in the fluorescence intensity as a function of the volume (50 to 200 µL) of the CN sensor that was introduced through the intranasal application into the lungs of the mice infected with PA14. All of the data are given as the mean ± s.d. of n independent measurements. (DOCX) [file pone.0021387.s003.docx]

**
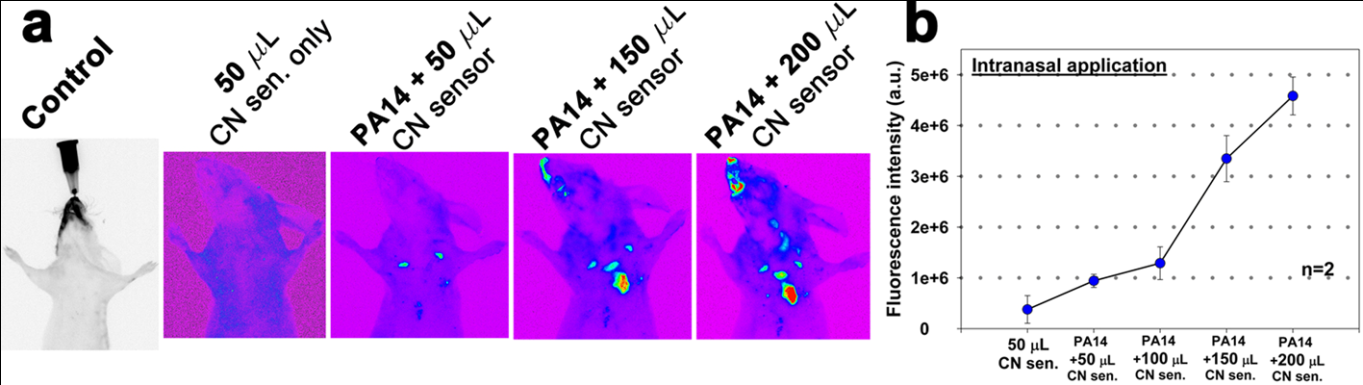
**

**Figure S3** *In vivo* imaging of CN in the lung of PA14-infected mice using the cyanide sensor with the intranasal application. (a) A 24-gauge catheter containing 50 μL of the cyanide sensor (1 mM) was introduced into the nostril of the mouse that was not infected with PA14 (control). 50 - 200 μL of the CN sensor (1 mM) was injected into the lung of PA14-infected mice through the intranasal application at 18 h. post infection. (b) Changes in the fluorescence intensity as a function of the volume (50 to 200 μL) of the CN sensor that was introduced through the intranasal application into the lungs of the mice infected with PA14. All of the data are given as the mean ± s.d. of n independent measurements.
