# Supplementary material for: In Vivo Fluorescence Imaging of Bacteriogenic Cyanide in the Lungs of Live Mice Infected with Cystic Fibrosis Pathogens
Source: PLoS One. 2011 Jul 7;6(7):e21387. doi: 10.1371/journal.pone.0021387 (PMC3131278; doi:10.1371/journal.pone.0021387)
Supplement: Figure S4 — Effect of the volume of the CN sensor on in vivo cyanide imaging. (a) Various volumes (0–100 µL) of the CN sensor (1 mM) were injected into the lung of mice infected with PA14. The images in the upper and lower panels are the inverted fluorescence images and their corresponding reconstructed color images, respectively. (b) Changes in the fluorescence intensity as a function of the injected volume of the cyanide sensor in the lung of mice infected with PA14. (c) Signal to noise (S/N) ratio of the fluorescence signal in the mouse lungs with respect to the injected volume of the cyanide sensor. The S/N ratio was defined as the ratio of the fluorescent signal value in the mouse lung infected with PA14 to the signal value without the PA14 infection. All of the data are given as the mean ± standard deviations (s.d.) of n independent measurements. (DOCX) [file pone.0021387.s004.docx]

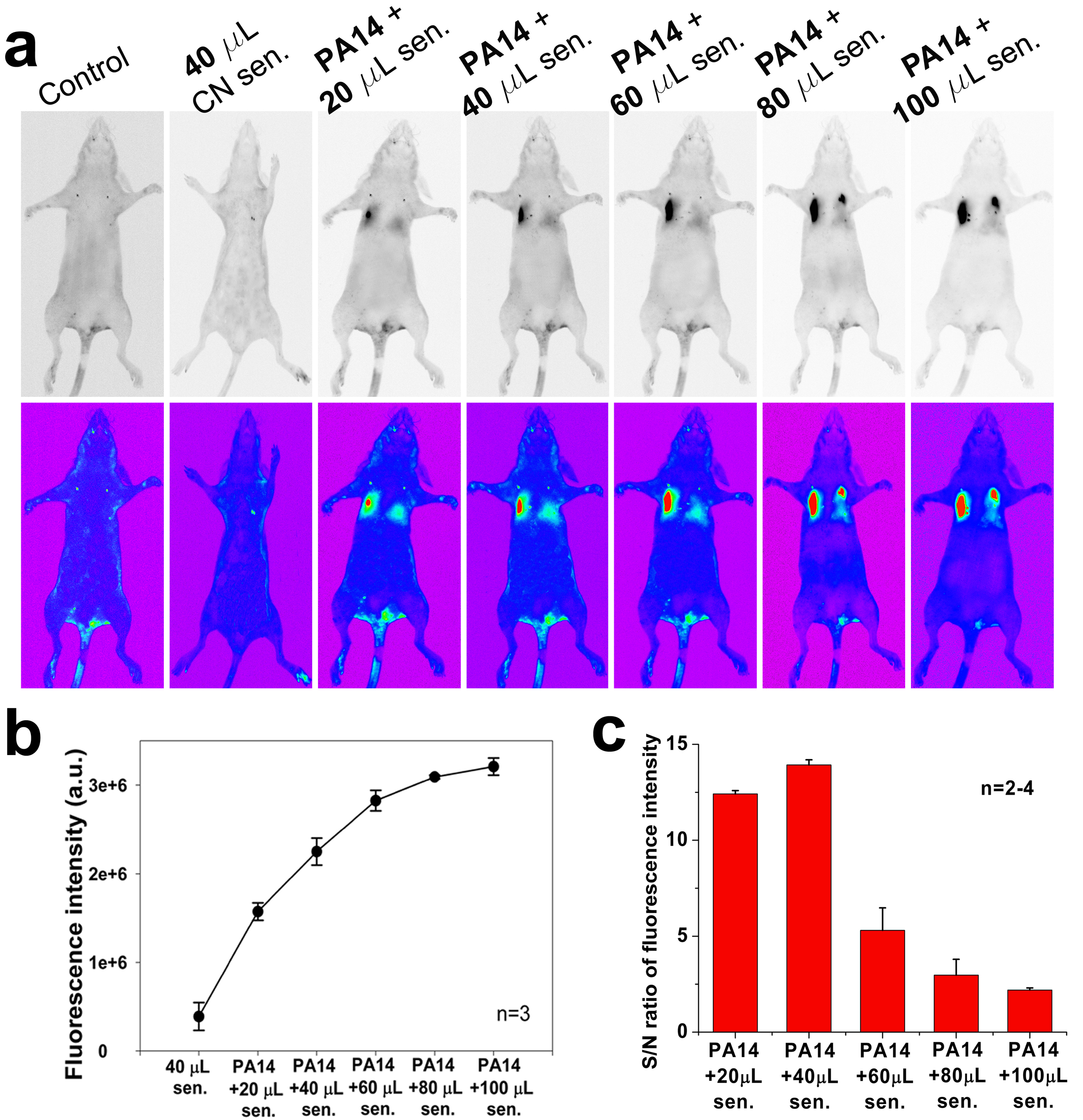


**Figure S4** Effect of the volume of the CN sensor on i*n vivo* cyanide imaging. (a) Various volumes (0-100 μL) of the CN sensor (1 mM) were injected into the lung of mice infected with PA14. The images in the upper and lower panels are the inverted fluorescence images and their corresponding reconstructed color images, respectively. (b) Changes in the fluorescence intensity as a function of the injected volume of the cyanide sensor in the lung of mice infected with PA14. (c) Signal to noise (S/N) ratio of the fluorescence signal in the mouse lungs with respect to the injected volume of the cyanide sensor. The S/N ratio was defined as the ratio of the fluorescent signal value in the mouse lung infected with PA14 to the signal value without the PA14 infection. All of the data are given as the mean ± standard deviations (s.d.) of n independent measurements.
