# Supplementary material for: In Vivo Fluorescence Imaging of Bacteriogenic Cyanide in the Lungs of Live Mice Infected with Cystic Fibrosis Pathogens
Source: PLoS One. 2011 Jul 7;6(7):e21387. doi: 10.1371/journal.pone.0021387 (PMC3131278; doi:10.1371/journal.pone.0021387)
Supplement: Figure S5 — Ex vivo imaging of CN and biofilm in the lungs of the mice infected with PA14 or B. cepacia strains for various times. CLSM images of the 20 µm thick cryo-sections incubated with 2 µM CN sensor for 5 min and 50 µg/mL concanavalin A-TRITC for 5 min. The surface plot image of merged images was obtained using Imaris™ program. Scale bar −20 µm. (DOCX) [file pone.0021387.s005.docx]

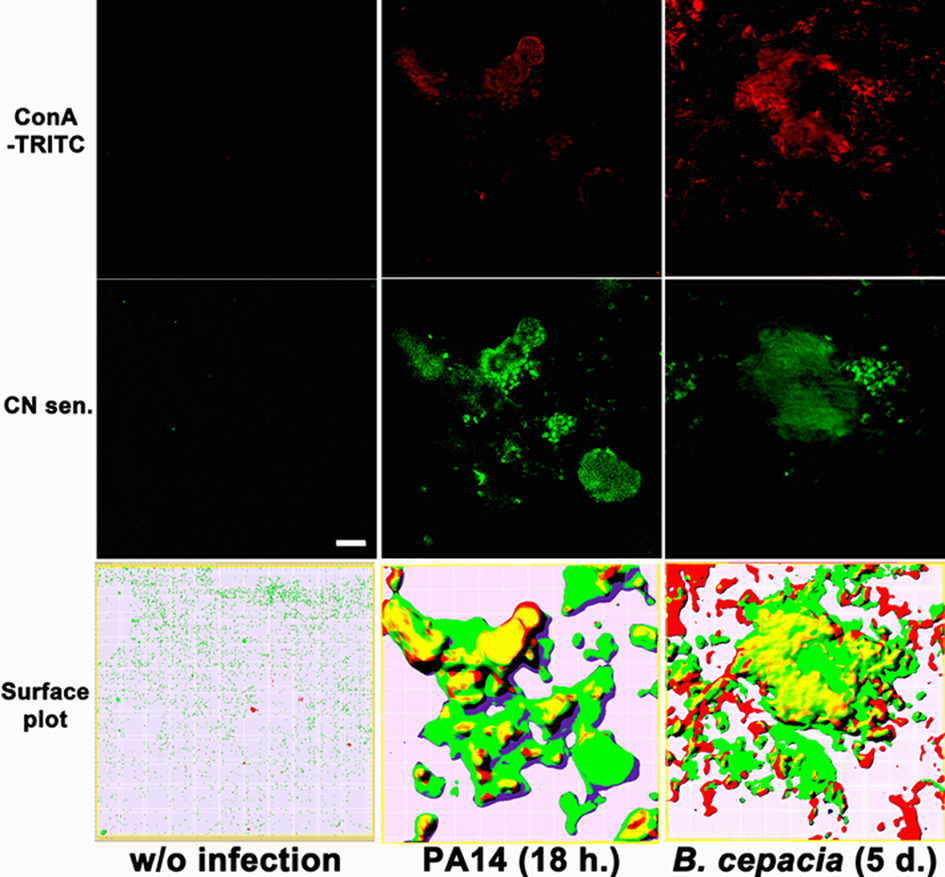


**Figure S5** *Ex vivo* imaging of CN and biofilm in the lungs of the mice infected with PA14 or *B. cepacia* strains for various times. CLSM images of the 20 μm thick cryo-sections incubated with 2 μM CN sensor for 5 min and 50 μg/mL concanavalin A-TRITC for 5 min. The surface plot image of merged images was obtained using Imaris™ program. Scale bar - 20 μm.
