# Supplementary material for: In Vivo Fluorescence Imaging of Bacteriogenic Cyanide in the Lungs of Live Mice Infected with Cystic Fibrosis Pathogens
Source: PLoS One. 2011 Jul 7;6(7):e21387. doi: 10.1371/journal.pone.0021387 (PMC3131278; doi:10.1371/journal.pone.0021387)
Supplement: Table S1 — CN production in liquid and solid cultures and murine lungs by PA and B. cepacia strains. CN in the liquid cultures was determined using the Spectroquant® method* in triplicate (at OD600 = 0.9 for PA, 2.6 for B. cepacia). CN in the solid cultures containing glass beads were trapped in 4 M NaOH and measured using the method described above. Means ± standard deviations of three independent experiments are shown (n. d. = not detected). Biogenic CN in the lungs previously infected with PA and B. cepacia was determined from the images using a multimodal-imaging system (IS4000MM, Kodak). The standard curve ( Fig. 2b ) was used to determine the concentration of biogenic CN in the lungs infected with either PA or B. cepacia. *Detailed description in the methods. (DOCX) [file pone.0021387.s006.docx]

| **Strain**  **(+ antibiotics)** | **Description** | **CN conc. in bacterial**  **culture (μM)** | **Estimated CN conc.**  **in the lung (mM)** | **References** |
| --- | --- | --- | --- | --- |
| **PAO1** | **Wild type,**  **prototrophic strain.** | **11.6 ± 2.2** | **1.7± 0.1** | **35** |
| **PA14** | **Clinical isolate**  **UCBPP-PA14.** | **42.4 ± 3.3** | **2.9 ± 0.1** | **34** |
| **PA14/*hcn*C** | **Mutant for HCN synthase (*hcnABC*).** | **0** | **0** | **36** |
| **PA14 + Ceftazidime** | **+ 200 mg/kg/day** | **n. d.** | **0.9 ± 0.1** | **27** |
| **PA14 + Ciprofloxacin** | **+ 30 mg/kg/day** | **n. d.** | **1.6 ± 0.3** | **27** |
| **PA14 + Patulin** | **+ 2.5 mg/kg/day**  **for 3 days** | **n. d.** | **0.5 ± 0.1** | **28** |
| ***B. cepacia*** | **In liquid culture** | **n. d.** | **4.0 ± 0.7** |  |
| ***B. cepacia*** | **Glass beads**  **biofilm assay*** | **197.4 ± 25.9** | **(5 days post infection)** | **8** |
| ***B. cepacia* + Ceftazidime** | **+ 200 mg/kg/day** | **0.2 ± 0.01**  **(18 h, 28°C)** | **3.4 ± 0.5** | **27** |
| ***B. cepacia* + Ciprofloxacin** | **+ 30 mg/kg/day** | **0.2 ± 0.1**  **(18 h, 28°C)** | **3.9 ± 0.8** | **27** |
| ***B. cepacia* + Patulin** | **+ 2.5 mg/mkg/day** | **0.2 ± 0.01**  **(18 h, 28°C)** | **3.6 ± 0.8** | **28** |

**Table S1** CN production in liquid and solid cultures and murine lungs by PA and *B. cepacia* strains. CN in the liquid cultures was determined using the Spectroquant^®^ method* in triplicate (at OD_600_ = 0.9 for PA, 2.6 for *B. cepacia*). CN in the solid cultures containing glass beads were trapped in 4 M NaOH and measured using the method described above. Means ± standard deviations of three independent experiments are shown (n. d. = not detected). Biogenic CN in the lungs previously infected with PA and *B. cepacia* was determined from the images using a multimodal-imaging system (IS4000MM, Kodak). The standard curve (**Fig. 2b**) was used to determine the concentration of biogenic CN in the lungs infected with either PA or *B. cepacia*. *Detailed description in the methods.
